# County, subregional and regional nitrogen data derived from the Net Anthropogenic Nitrogen Inputs (NANI) toolbox

**DOI:** 10.1016/j.dib.2018.04.098

**Published:** 2018-05-02

**Authors:** Dennis P. Swaney, Robert W. Howarth, Bongghi Hong

**Affiliations:** Cornell University, United States

## Abstract

[The data presented here represent estimates of the nitrogen content of crop production, nitrogen use efficiency (NUE) and agricultural nitrogen inputs associated with it across the contiguous United States. Net Anthropogenic Nitrogen Input (NANI) estimates and related data are also provided. Data are presented at county, sub-regional and regional scales. Here, subregions refer to multi-county areas delineated with the goal of obtaining more uniform reporting areas than individual counties. Regions refer to the USDA Farm Resource Regions. The data are reported for 6 agricultural census years, 1987, 1992, 1997, 2002, 2007 and 2012. Estimates of the variables were derived originally from USDA agricultural census data, US population census data, and other sources, using version 3.1 of the NANI calculator toolbox [1], [2], [3]].

**Specifications table**

**Table:** 

Subject area	*Biogeochemistry, agriculture, ecology*
More specific subject area	*Nitrogen cycling, nitrogen use efficiency, net anthropogenic nitrogen inputs (NANI)*
Type of data	*Tables, multi-page spreadsheet*
How data was acquired	*Derived from publicly available sources, including US Census of Agriculture, US population Census, and processed using the NANI toolbox*
Data format	*Processed and analyzed*
Experimental factors	–
Experimental features	–
Data source location	*Continental United States*
Data accessibility	*Source data from which these data are derived were obtained from the NANI toolbox, Swaney, D.P., Hong, B., and Howarth, R.W. 2018. NANI/NAPI Calculator Toolbox Version 3.1 Documentation. http://www.eeb.cornell.edu/biogeo/nanc/nani/NANI NAPI_Calculator_Toolbox_Version_3.1_Documentation.docx*
Related research article	*Swaney, D.P., R.W. Howarth, and B. Hong. Nitrogen use efficiency and crop production: Patterns of regional variation in the United States, 1987-2012. In Press. Science of the Total Environment.*

**Value of the data**

• These data represent regional variation of major agricultural nitrogen flows across the US over 3 decades at 5 year intervals.
• As such, they may be useful for other studies of regional variation in a biogeochemical, ecological or agricultural context.
• The data are of sufficient resolution (county and multi-county) that they may also be useful for within-region or large-watershed studies.

## 1 Data

A full list of the variables provided at county, subregional scales, together with a short description of the variables and their units are provided in Table 1, Table 2, Table 3. Details on the characteristics of the regions and subregions considered are provided in Table 1 of [4], together with maps of regions (Figure 1) and subregions (Figure S1) in the same paper.

**Table 1:** Description of variables provided at the county-scale.

**Variable name**	**Units/Format**	**Description**
FIPScode	Number	Federal Information Processing Standard county code
County Name	Text	County Name
State	Text	State Name
State Code	Text	State Code
Subregion Code	Text	Subregion Code
Region Name	Text	Region Name
Region Code	Text	Region Code
NANI 1987	kg N km^−2^ yr^−1^	Net anthropogenic nitrogen inputs to counties, 1987
NANI 1992	kg N km^−2^ yr^−1^	Net anthropogenic nitrogen inputs to counties, 1992
NANI 1997	kg N km^−2^ yr^−1^	Net anthropogenic nitrogen inputs to counties, 1997
NANI 2002	kg N km^−2^ yr^−1^	Net anthropogenic nitrogen inputs to counties, 2002
NANI 2007	kg N km^−2^ yr^−1^	Net anthropogenic nitrogen inputs to counties, 2007
NANI 2012	kg N km^−2^ yr^−1^	Net anthropogenic nitrogen inputs to counties, 2012
NFF 1987	kg N km^−2^ yr−^1^	Net food/feed nitrogen inputs to counties, 1987
NFF 1992	kg N km^−2^ yr^−1^	Net food/feed nitrogen inputs to counties, 1992
NFF 1997	kg N km^−2^ yr^−1^	Net food/feed nitrogen inputs to counties, 1997
NFF 2002	kg N km^−2^ yr^−1^	Net food/feed nitrogen inputs to counties, 2002
NFF 2007	kg N km^−2^ yr^−1^	Net food/feed nitrogen inputs to counties, 2007
NFF 2012	kg N km^−2^ yr^−1^	Net food/feed nitrogen inputs to counties, 2012
NFERT 1987	kg N km^−2^ yr^−1^	Total fertilizer nitrogen inputs to counties, 1987
NFERT 1992	kg N km^−2^ yr^−1^	Total fertilizer nitrogen inputs to counties, 1992
NFERT 1997	kg N km^−2^ yr^−1^	Total fertilizer nitrogen inputs to counties, 1997
NFERT 2002	kg N km^−2^ yr^−1^	Total fertilizer nitrogen inputs to counties, 2002
NFERT 2007	kg N km^−2^ yr^−1^	Total fertilizer nitrogen inputs to counties, 2007
NFERT 2012	kg N km^−2^ yr^−1^	Total fertilizer nitrogen inputs to counties, 2012
AG NFERT 1987	kg N km^−2^ yr^−1^	Agricultural fertilizer nitrogen inputs to counties, 1987
AG NFERT 1992	kg N km^−2^ yr^−1^	Agricultural fertilizer nitrogen inputs to counties, 1992
AG NFERT 1997	kg N km^−2^ yr^−1^	Agricultural fertilizer nitrogen inputs to counties, 1997
AG NFERT 2002	kg N km^−2^ yr^−1^	Agricultural fertilizer nitrogen inputs to counties, 2002
AG NFERT 2007	kg N km^−2^ yr^−1^	Agricultural fertilizer nitrogen inputs to counties, 2007
AG NFERT 2012	kg N km^−2^ yr^−1^	Agricultural fertilizer nitrogen inputs to counties, 2012
NOY DEP 1987	kg N km^−2^ yr^−1^	Oxidized nitrogen atmospheric N inputs to counties, 1987
NOY DEP 1992	kg N km^−2^ yr^−1^	Oxidized nitrogen atmospheric N inputs to counties, 1992
NOY DEP 1997	kg N km^−2^ yr^−1^	Oxidized nitrogen atmospheric N inputs to counties, 1997
NOY DEP 2002	kg N km^−2^ yr^−1^	Oxidized nitrogen atmospheric N inputs to counties, 2002
NOY DEP 2007**	kg N km^−2^ yr^−1^	Oxidized nitrogen atmospheric N inputs to counties, 2007
NOY DEP 2012**	kg N km^−2^ yr^−1^	Oxidized nitrogen atmospheric N inputs to counties, 2012
NFIX 1987	kg N km^−2^ yr^−1^	Crop N fixation per area of county, 1987
NFIX 1992	kg N km^−2^ yr^−1^	Crop N fixation per area of county, 1992
NFIX 1997	kg N km^−2^ yr^−1^	Crop N fixation per area of county, 1997
NFIX 2002	kg N km^−2^ yr^−1^	Crop N fixation per area of county, 2002
NFIX 2007	kg N km^−2^ yr^−1^	Crop N fixation per area of county, 2007
NFIX 2012	kg N km^−2^ yr^−1^	Crop N fixation per area of county, 2012
County area	km^2^	County area
TOTAL CROPLAND AREA 1987	km^2^	Total cropland area, 1987
TOTAL CROPLAND AREA 1992	km^2^	Total cropland area, 1992
TOTAL CROPLAND AREA 1997	km^2^	Total cropland area, 1997
TOTAL CROPLAND AREA 2002	km^2^	Total cropland area, 2002
TOTAL CROPLAND AREA 2007	km^2^	Total cropland area, 2007
TOTAL CROPLAND AREA 2012	km^2^	Total cropland area, 2012
Y’, CROP N/TOT AREA 1987	kg N km^−2^ yr^−1^	N content of crops per area of county, 1987
Y’, CROP N/TOT AREA 1992	kg N km^−2^ yr^−1^	N content of crops per area of county, 1992
Y’, CROP N/TOT AREA 1997	kg N km^−2^ yr^−1^	N content of crops per area of county, 1997
Y’, CROP N/TOT AREA 2002	kg N km^−2^ yr^−1^	N content of crops per area of county, 2002
Y’, CROP N/TOT AREA 2007	kg N km^−2^ yr^−1^	N content of crops per area of county, 2007
Y’, CROP N/TOT AREA 2012	kg N km^−2^ yr^−1^	N content of crops per area of county, 2012
Y, CROP N/CROPLAND AREA 1987	kg N km^−2^ yr^−1^*	N content of crops per total cropland area, 1987
Y, CROP N/CROPLAND AREA 1992	kg N km^−2^ yr^−1^*	N content of crops per total cropland area, 1992
Y, CROP N/CROPLAND AREA 1997	kg N km^−2^ yr^−1^*	N content of crops per total cropland area, 1997
Y, CROP N/CROPLAND AREA 2002	kg N km^−2^ yr^−1^*	N content of crops per total cropland area, 2002
Y, CROP N/CROPLAND AREA 2007	kg N km^−2^ yr^−1^*	N content of crops per total cropland area, 2007
Y, CROP N/CROPLAND AREA 2012	kg N km^−2^ yr^−1^*	N content of crops per total cropland area, 2012
AG NFERT/CROPLAND AREA, 1987	kg N km^−2^ yr^−1^*	Agricultural fertilizer nitrogen inputs to counties per area of total cropland, 1987
AG NFERT/CROPLAND AREA, 1992	kg N km^−2^ yr^−1^*	Agricultural fertilizer nitrogen inputs to counties per area of total cropland, 1992
AG NFERT/CROPLAND AREA, 1997	kg N km^−2^ yr^−1^*	Agricultural fertilizer nitrogen inputs to counties per area of total cropland, 1997
AG NFERT/CROPLAND AREA, 2002	kg N km^−2^ yr^−1^*	Agricultural fertilizer nitrogen inputs to counties per area of total cropland, 2002
AG NFERT/CROPLAND AREA, 2007	kg N km^−2^ yr^−1^*	Agricultural fertilizer nitrogen inputs to counties per area of total cropland, 2007
AG NFERT/CROPLAND AREA, 2012	kg N km^−2^ yr^−1^*	Agricultural fertilizer nitrogen inputs to counties per area of total cropland, 2012
**Variable Name**	**Units/Format**	**Description**
Pop, 1987	Individuals	County population, 1987
Pop, 1992	Individuals	County population, 1992
Pop, 1997	Individuals	County population, 1997
Pop, 2002	Individuals	County population, 2002
Pop, 2007	Individuals	County population, 2007
Pop, 2012	Individuals	County population, 2012
NFIX/CROPLAND AREA 1987	kg N km^−2^ yr^−1^*	Crop N fixation per total cropland area, 1987
NFIX/CROPLAND AREA 1992	kg N km^−2^ yr^−1^*	Crop N fixation per total cropland area, 1992
NFIX/CROPLAND AREA 1997	kg N km^−2^ yr^−1^*	Crop N fixation per total cropland area, 1997
NFIX/CROPLAND AREA 2002	kg N km^−2^ yr^−1^*	Crop N fixation per total cropland area, 2002
NFIX/CROPLAND AREA 2007	kg N km^−2^ yr^−1^*	Crop N fixation per total cropland area, 2007
NFIX/CROPLAND AREA 2012	kg N km^−2^ yr^−1^*	Crop N fixation per total cropland area, 2012
NEXCR/TOT AREA 1987	kg N km^−2^ yr^−1^	Livestock N excretion per total county area, 1987
NEXCR/TOT AREA 1992	kg N km^−2^ yr^−1^	Livestock N excretion per total county area, 1992
NEXCR/TOT AREA 1997	kg N km^−2^ yr^−1^	Livestock N excretion per total county area, 1997
NEXCR/TOT AREA 2002	kg N km^−2^ yr^−1^	Livestock N excretion per total county area, 2002
NEXCR/TOT AREA 2007	kg N km^−2^ yr^−1^	Livestock N excretion per total county area, 2007
NEXCR/TOT AREA 2012	kg N km^−2^ yr^−1^	Livestock N excretion per total county area, 2012
NEXCR/CROPLAND AREA 1987	kg N km^−2^ yr^−1^*	Livestock N excretion per total cropland area, 1987
NEXCR/CROPLAND AREA 1992	kg N km^−2^ yr^−1^*	Livestock N excretion per total cropland area, 1992
NEXCR/CROPLAND AREA 1997	kg N km^−2^ yr^−1^*	Livestock N excretion per total cropland area, 1997
NEXCR/CROPLAND AREA 2002	kg N km^−2^ yr^−1^*	Livestock N excretion per total cropland area, 2002
NEXCR/CROPLAND AREA 2007	kg N km^−2^ yr^−1^*	Livestock N excretion per total cropland area, 2007
NEXCR/CROPLAND AREA 2012	kg N km^−2^ yr^−1^*	Livestock N excretion per total cropland area, 2012
I, 1987	kg N km^−2^ yr^−1^*	Total ag N inputs per cropland area, 1987
I, 1992	kg N km^−2^ yr^−1^*	Total ag N inputs per cropland area, 1992
I, 1997	kg N km^−2^ yr^−1^*	Total ag N inputs per cropland area, 1997
I, 2002	kg N km^−2^ yr^−1^*	Total ag N inputs per cropland area, 2002
I, 2007	kg N km^−2^ yr^−1^*	Total ag N inputs per cropland area, 2007
I, 2012	kg N km^−2^ yr^−1^*	Total ag N inputs per cropland area, 2012
I’, 1987	kg N km^−2^ yr^−1^	Total ag N inputs per county area, 1987
I’, 1992	kg N km^−2^ yr^−1^	Total ag N inputs per county area, 1992
I’, 1997	kg N km^−2^ yr^−1^	Total ag N inputs per county area, 1997
I’, 2002	kg N km^−2^ yr^−1^	Total ag N inputs per county area, 2002
I’, 2007	kg N km^−2^ yr^−1^	Total ag N inputs per county area, 2007
I’, 2012	kg N km^−2^ yr^−1^	Total ag N inputs per county area, 2012
**Variable Name**	**Units/Format**	**Description**
NUE, 1987	%	Nitrogen Use Efficiency, Y/I, 1987, as %
NUE, 1992	%	Nitrogen Use Efficiency, Y/I, 1992, as %
NUE, 1997	%	Nitrogen Use Efficiency, Y/I, 1997, as %
NUE, 2002	%	Nitrogen Use Efficiency, Y/I, 2002, as %
NUE, 2007	%	Nitrogen Use Efficiency, Y/I, 2007, as %
NUE, 2012	%	Nitrogen Use Efficiency, Y/I, 2012, as %
Nfert%, 1987	%	Ag N fertilizer as % of total county N input, 1987
Nfert%, 1992	%	Ag N fertilizer as % of total county N input, 1992
Nfert%, 1997	%	Ag N fertilizer as % of total county N input, 1997
Nfert%, 2002	%	Ag N fertilizer as % of total county N input, 2002
Nfert%, 2007	%	Ag N fertilizer as % of total county N input, 2007
Nfert%, 2012	%	Ag N fertilizer as % of total county N input, 2012
Nfix%, 1987	%	Crop N fixation as % of total county N input, 1987
Nfix%, 1992	%	Crop N fixation as % of total county N input, 1992
Nfix%, 1997	%	Crop N fixation as % of total county N input, 1997
Nfix%, 2002	%	Crop N fixation as % of total county N input, 2002
Nfix%, 2007	%	Crop N fixation as % of total county N input, 2007
Nfix%, 2012	%	Crop N fixation as % of total county N input, 2012
Nexcr%, 1987	%	Livestock N excr. % of total county N input, 1987
Nexcr%, 1992	%	Livestock N excr. % of total county N input, 1992
Nexcr%, 1997	%	Livestock N excr. % of total county N input, 1997
Nexcr%, 2002	%	Livestock N excr. % of total county N input, 2002
Nexcr%, 2007	%	Livestock N excr. % of total county N input, 2007
Nexcr%, 2012	%	Livestock N excr. % of total county N input, 2012
LP 1987***	kg N km^−2^ yr^−1^	Production (N) of livestock products in counties, 1987
LP 1992***	kg N km^−2^ yr^−1^	Production (N) of livestock products in counties, 1992
LP 1997***	kg N km^−2^ yr^−1^	Production (N) of livestock products in counties, 1997
LP 2002***	kg N km^−2^ yr^−1^	Production (N) of livestock products in counties, 2002
LP 2007***	kg N km^−2^ yr^−1^	Production (N) of livestock products in counties, 2007
LP 2012***	kg N km^−2^ yr^−1^	Production (N) of livestock products in counties, 2012

* Indicates cropland area basis instead of total area basis

** N deposition estimate is from the most recent available year of CMAQ model estimates (2006); [1]

*** Beef & veal, pork, poultry, lamb, eggs, milk

**Table 2:** Description of variables provided at the subregional scale.

**Variable name**	**Units/Format**	**Description**	
Subregion Code	Text	Subregion Code	
Region Name	Text	Region Name	
Region Code	Text	Region Code	
NANI 1987	kg N km^−2^ yr^−1^	Net anthropogenic nitrogen inputs to subregions, 1987	
NANI 1992	kg N km^−2^ yr^−1^	Net anthropogenic nitrogen inputs to subregions, 1992	
NANI 1997	kg N km^−2^ yr^−1^	Net anthropogenic nitrogen inputs to subregions, 1997	
NANI 2002	kg N km^−2^ yr^−1^	Net anthropogenic nitrogen inputs to subregions, 2002	
NANI 2007	kg N km^−2^ yr^−1^	Net anthropogenic nitrogen inputs to subregions, 2007	
NANI 2012	kg N km^−2^ yr^−1^	Net anthropogenic nitrogen inputs to subregions, 2012	
NFF 1987	kg N km^−2^ yr^−1^	Net food/feed nitrogen inputs to subregions, 1987	
NFF 1992	kg N km^−2^ yr^−1^	Net food/feed nitrogen inputs to subregions, 1992	
NFF 1997	kg N km^−2^ yr^−1^	Net food/feed nitrogen inputs to subregions, 1997	
NFF 2002	kg N km^−2^ yr^−1^	Net food/feed nitrogen inputs to subregions, 2002	
NFF 2007	kg N km^−2^ yr^−1^	Net food/feed nitrogen inputs to subregions, 2007	
NFF 2012	kg N km^−2^ yr^−1^	Net food/feed nitrogen inputs to subregions, 2012	
NFERT 1987	kg N km^−2^ yr^−1^	Total fertilizer nitrogen inputs to subregions, 1987	
NFERT 1992	kg N km^−2^ yr^−1^	Total fertilizer nitrogen inputs to subregions, 1992	
NFERT 1997	kg N km^−2^ yr^−1^	Total fertilizer nitrogen inputs to subregions, 1997	
NFERT 2002	kg N km^−2^ yr^−1^	Total fertilizer nitrogen inputs to subregions, 2002	
NFERT 2007	kg N km^−2^ yr^−1^	Total fertilizer nitrogen inputs to subregions, 2007	
NFERT 2012	kg N km^−2^ yr^−1^	Total fertilizer nitrogen inputs to subregions, 2012	
AG NFERT 1987	kg N km^−2^ yr^−1^	Agricultural fertilizer nitrogen inputs to subregions, 1987	
AG NFERT 1992	kg N km^−2^ yr^−1^	Agricultural fertilizer nitrogen inputs to subregions, 1992	
AG NFERT 1997	kg N km^−2^ yr^−1^	Agricultural fertilizer nitrogen inputs to subregions, 1997	
AG NFERT 2002	kg N km^−2^ yr^−1^	Agricultural fertilizer nitrogen inputs to subregions, 2002	
AG NFERT 2007	kg N km^−2^ yr^−1^	Agricultural fertilizer nitrogen inputs to subregions, 2007	
AG NFERT 2012	kg N km^−2^ yr^−1^	Agricultural fertilizer nitrogen inputs to subregions, 2012	
NOY DEP 1987	kg N km^−2^ yr^−1^	Oxidized nitrogen atmospheric N inputs to subregions, 1987	
NOY DEP 1992	kg N km^−2^ yr^−1^	Oxidized nitrogen atmospheric N inputs to subregions, 1992	
NOY DEP 1997	kg N km^−2^ yr^−1^	Oxidized nitrogen atmospheric N inputs to subregions, 1997	
NOY DEP 2002	kg N km^−2^ yr^−1^	Oxidized nitrogen atmospheric N inputs to subregions, 2002	
NOY DEP 2007**	kg N km^−2^ yr^−1^	Oxidized nitrogen atmospheric N inputs to subregions, 2007	
NOY DEP 2012**	kg N km^−2^ yr^−1^	Oxidized nitrogen atmospheric N inputs to subregions, 2012	
NFIX 1987	kg N km^−2^ yr^−1^	Crop N fixation per area of subregion, 1987	
NFIX 1992	kg N km^−2^ yr^−1^	Crop N fixation per area of subregion, 1992	
NFIX 1997	kg N km^−2^ yr^−1^	Crop N fixation per area of subregion, 1997	
NFIX 2002	kg N km^−2^ yr^−1^	Crop N fixation per area of subregion, 2002	
NFIX 2007	kg N km^−2^ yr^−1^	Crop N fixation per area of subregion, 2007	
NFIX 2012	kg N km^−2^ yr^−1^	Crop N fixation per area of subregion, 2012	
Subregion area	km^2^		Subregion area
TOTAL CROPLAND AREA 1987	km^2^		Total subregional cropland area, 1987
TOTAL CROPLAND AREA 1992	km^2^		Total subregional cropland area, 1992
TOTAL CROPLAND AREA 1997	km^2^		Total subregional cropland area, 1997
TOTAL CROPLAND AREA 2002	km^2^		Total subregional cropland area, 2002
TOTAL CROPLAND AREA 2007	km^2^		Total subregional cropland area, 2007
TOTAL CROPLAND AREA 2012	km^2^		Total subregional cropland area, 2012
Y’, CROP N/TOT AREA 1987	kg N km^−2^ yr^−1^		N content of crops per area of subregion, 1987
Y’, CROP N/TOT AREA 1992	kg N km^−2^ yr^−1^		N content of crops per area of subregion, 1992
Y’, CROP N/TOT AREA 1997	kg N km^−2^ yr^−1^		N content of crops per area of subregion, 1997
Y’, CROP N/TOT AREA 2002	kg N km^−2^ yr^−1^		N content of crops per area of subregion, 2002
Y’, CROP N/TOT AREA 2007	kg N km^−2^ yr^−1^		N content of crops per area of subregion, 2007
Y’, CROP N/TOT AREA 2012	kg N km^−2^ yr^−1^		N content of crops per area of subregion, 2012
Y, CROP N/CROPLAND AREA 1987	kg N km^−2^ yr^−1^*		N content of crops per subregional cropland area, 1987
Y, CROP N/CROPLAND AREA 1992	kg N km^−2^ yr^−1^*		N content of crops per subregional cropland area, 1992
Y, CROP N/CROPLAND AREA 1997	kg N km^−2^ yr^−1^*		N content of crops per subregional cropland area, 1997
Y, CROP N/CROPLAND AREA 2002	kg N km^−2^ yr^−1^*		N content of crops per subregional cropland area, 2002
Y, CROP N/CROPLAND AREA 2007	kg N km^−2^ yr^−1^*		N content of crops per subregional cropland area, 2007
Y, CROP N/CROPLAND AREA 2012	kg N km^−2^ yr^−1^*		N content of crops per subregional cropland area, 2012
AG NFERT/CROPLAND AREA, 1987	kg N km^−2^ yr^−1^*		Agricultural fertilizer nitrogen inputs to subregions per area of subregional cropland, 1987
AG NFERT/CROPLAND AREA, 1992	kg N km^−2^ yr^-1^*		Agricultural fertilizer nitrogen inputs to subregions per area of subregional cropland, 1992
AG NFERT/CROPLAND AREA, 1997	kg N km^−2^ yr^-1^*		Agricultural fertilizer nitrogen inputs to subregions per area of subregional cropland, 1997
AG NFERT/CROPLAND AREA, 2002	kg N km^−2^ yr^−1^*		Agricultural fertilizer nitrogen inputs to subregions per area of subregional cropland, 2002
AG NFERT/CROPLAND AREA, 2007	kg N km^−2^ yr^−1^*		Agricultural fertilizer nitrogen inputs to subregions per area of subregional cropland, 2007
AG NFERT/CROPLAND AREA, 2012	kg N km^−2^ yr^−1^*		Agricultural fertilizer nitrogen inputs to subregions per area of subregional cropland, 2012
Pop, 1987	Individuals		Subregional population, 1987
Pop, 1992	Individuals		Subregional population, 1992
Pop, 1997	Individuals		Subregional population, 1997
Pop, 2002	Individuals		Subregional population, 2002
Pop, 2007	Individuals		Subregional population, 2007
Pop, 2012	Individuals		Subregional population, 2012
NFIX/CROPLAND AREA 1987	kg N km^−2^ yr^−1^*		Crop N fixation per subregional cropland area, 1987
NFIX/CROPLAND AREA 1992	kg N km^−2^ yr^−1^*		Crop N fixation per subregional cropland area, 1992
NFIX/CROPLAND AREA 1997	kg N km^−2^ yr^−1^*		Crop N fixation per subregional cropland area, 1997
NFIX/CROPLAND AREA 2002	kg N km^−2^ yr^−1^*		Crop N fixation per subregional cropland area, 2002
NFIX/CROPLAND AREA 2007	kg N km^−2^ yr^−1^*		Crop N fixation per subregional cropland area, 2007
NFIX/CROPLAND AREA 2012	kg N km^−2^ yr^−1^*		Crop N fixation per subregional cropland area, 2012
NEXCR/TOT AREA 1987	kg N km^−2^ yr^−1^		Livestock N excretion per total subregion area, 1987
NEXCR/TOT AREA 1992	kg N km^−2^ yr^−1^		Livestock N excretion per total subregion area, 1992
NEXCR/TOT AREA 1997	kg N km^−2^ yr^−1^		Livestock N excretion per total subregion area, 1997
NEXCR/TOT AREA 2002	kg N km^−2^ yr^−1^		Livestock N excretion per total subregion area, 2002
NEXCR/TOT AREA 2007	kg N km^−2^ yr^−1^		Livestock N excretion per total subregion area, 2007
NEXCR/TOT AREA 2012	kg N km^−2^ yr^−1^		Livestock N excretion per total subregion area, 2012
NEXCR/CROPLAND AREA 1987	kg N km^−2^ yr^−1^*		Livestock N excretion per subregional cropland area, 1987
NEXCR/CROPLAND AREA 1992	kg N km^-2^ yr^-1^*		Livestock N excretion per subregional cropland area, 1992
NEXCR/CROPLAND AREA 1997	kg N km^-2^ yr^-1^*		Livestock N excretion per subregional cropland area, 1997
NEXCR/CROPLAND AREA 2002	kg N km^-2^ yr^-1^*		Livestock N excretion per subregional cropland area, 2002
NEXCR/CROPLAND AREA 2007	kg N km^-2^ yr^-1^*		Livestock N excretion per subregional cropland area, 2007
NEXCR/CROPLAND AREA 2012	kg N km^-2^ yr^-1^*		Livestock N excretion per subregional cropland area, 2012
I, 1987	kg N km^-2^ yr^-1^*		Total ag N inputs per cropland area, 1987
I, 1992	kg N km^-2^ yr^-1^*		Total ag N inputs per cropland area, 1992
I, 1997	kg N km^-2^ yr^-1^*		Total ag N inputs per cropland area, 1997
I, 2002	kg N km^-2^ yr^-1^*		Total ag N inputs per cropland area, 2002
I, 2007	kg N km^-2^ yr^-1^*		Total ag N inputs per cropland area, 2007
I, 2012	kg N km^-2^ yr^-1^*		Total ag N inputs per cropland area, 2012
I’, 1987	kg N km^-2^ yr^-1^		Total ag N inputs per subregion area, 1987
I’, 1992	kg N km^-2^ yr^-1^		Total ag N inputs per subregion area, 1992
I’, 1997	kg N km^-2^ yr^-1^		Total ag N inputs per subregion area, 1997
I’, 2002	kg N km^-2^ yr^-1^		Total ag N inputs per subregion area, 2002
I’, 2007	kg N km^-2^ yr^-1^		Total ag N inputs per subregion area, 2007
I’, 2012	kg N km^-2^ yr^-1^		Total ag N inputs per subregion area, 2012
NUE, 1987	%		Nitrogen Use Efficiency, Y/I, 1987, as %
NUE, 1992	%		Nitrogen Use Efficiency, Y/I, 1992, as %
NUE, 1997	%		Nitrogen Use Efficiency, Y/I, 1997, as %
NUE, 2002	%		Nitrogen Use Efficiency, Y/I, 2002, as %
NUE, 2007	%		Nitrogen Use Efficiency, Y/I, 2007, as %
NUE, 2012	%		Nitrogen Use Efficiency, Y/I, 2012, as %
Nfert%, 1987	%		Ag N fertilizer as % of total subregional N input, 1987
Nfert%, 1992	%		Ag N fertilizer as % of total subregional N input, 1992
Nfert%, 1997	%		Ag N fertilizer as % of total subregional N input, 1997
Nfert%, 2002	%		Ag N fertilizer as % of total subregional N input, 2002
Nfert%, 2007	%		Ag N fertilizer as % of total subregional N input, 2007
Nfert%, 2012	%		Ag N fertilizer as % of total subregional N input, 2012
Nfix%, 1987	%		Crop N fixation as % of total subregional N input, 1987
Nfix%, 1992	%		Crop N fixation as % of total subregional N input, 1992
Nfix%, 1997	%		Crop N fixation as % of total subregional N input, 1997
Nfix%, 2002	%		Crop N fixation as % of total subregional N input, 2002
Nfix%, 2007	%		Crop N fixation as % of total subregional N input, 2007
Nfix%, 2012	%		Crop N fixation as % of total subregional N input, 2012
Nexcr%, 1987	%		Livestock N excr. % of total subregional N input, 1987
Nexcr%, 1992	%		Livestock N excr. % of total subregional N input, 1992
Nexcr%, 1997	%		Livestock N excr. % of total subregional N input, 1997
Nexcr%, 2002	%		Livestock N excr. % of total subregional N input, 2002
Nexcr%, 2007	%		Livestock N excr. % of total subregional N input, 2007
Nexcr%, 2012	%		Livestock N excr. % of total subregional N input, 2012
LP 1987***	kg N km^-2^ yr^-1^		Production (N) of livestock products in subregions, 1987
LP 1992***	kg N km^-2^ yr^-1^		Production (N) of livestock products in subregions, 1992
LP 1997***	kg N km^-2^ yr^-1^		Production (N) of livestock products in subregions, 1997
LP 2002***	kg N km^-2^ yr^-1^		Production (N) of livestock products in subregions, 2002
LP 2007***	kg N km^-2^ yr^-1^		Production (N) of livestock products in subregions, 2007
LP 2012***	kg N km^-2^ yr^-1^		Production (N) of livestock products in subregions, 2012

* Indicates cropland area basis instead of total area basis

** N deposition estimate is from the most recent available year of CMAQ model estimates (2006); [1]

*** Beef & veal, pork, poultry, lamb, eggs, milk

**Table 3:** Description of variables provided at the regional scale.

**Variable name**	**Units/Format**	**Description**
Region Name	Text	Region Name
Region Code	Text	Region Code
NANI 1987	kg N km^−2^ yr^−1^	Net anthropogenic nitrogen inputs to regions, 1987
NANI 1992	kg N km^−2^ yr^−1^	Net anthropogenic nitrogen inputs to regions, 1992
NANI 1997	kg N km^−2^ yr^−1^	Net anthropogenic nitrogen inputs to regions, 1997
NANI 2002	kg N km^−2^ yr^−1^	Net anthropogenic nitrogen inputs to regions, 2002
NANI 2007	kg N km^−2^ yr^−1^	Net anthropogenic nitrogen inputs to regions, 2007
NANI 2012	kg N km^−2^ yr^−1^	Net anthropogenic nitrogen inputs to regions, 2012
NFF 1987	kg N km^−2^ yr^−1^	Net food/feed nitrogen inputs to regions, 1987
NFF 1992	kg N km^−2^ yr^−1^	Net food/feed nitrogen inputs to regions, 1992
NFF 1997	kg N km^−2^ yr^−1^	Net food/feed nitrogen inputs to regions, 1997
NFF 2002	kg N km^−2^ yr^−1^	Net food/feed nitrogen inputs to regions, 2002
NFF 2007	kg N km^−2^ yr^−1^	Net food/feed nitrogen inputs to regions, 2007
NFF 2012	kg N km^−2^ yr^−1^	Net food/feed nitrogen inputs to regions, 2012
NFERT 1987	kg N km^−2^ yr^−1^	Total fertilizer nitrogen inputs to regions, 1987
NFERT 1992	kg N km^−2^ yr^−1^	Total fertilizer nitrogen inputs to regions, 1992
NFERT 1997	kg N km^−2^ yr^−1^	Total fertilizer nitrogen inputs to regions, 1997
NFERT 2002	kg N km^−2^ yr^−1^	Total fertilizer nitrogen inputs to regions, 2002
NFERT 2007	kg N km^−2^ yr^−1^	Total fertilizer nitrogen inputs to regions, 2007
NFERT 2012	kg N km^−2^ yr^−1^	Total fertilizer nitrogen inputs to regions, 2012
AG NFERT 1987	kg N km^−2^ yr^−1^	Agricultural fertilizer nitrogen inputs to regions, 1987
AG NFERT 1992	kg N km^−2^ yr^−1^	Agricultural fertilizer nitrogen inputs to regions, 1992
AG NFERT 1997	kg N km^−2^ yr^−1^	Agricultural fertilizer nitrogen inputs to regions, 1997
AG NFERT 2002	kg N km^−2^ yr^−1^	Agricultural fertilizer nitrogen inputs to regions, 2002
AG NFERT 2007	kg N km^−2^ yr^−1^	Agricultural fertilizer nitrogen inputs to regions, 2007
AG NFERT 2012	kg N km^−2^ yr^−1^	Agricultural fertilizer nitrogen inputs to regions, 2012
NOY DEP 1987	kg N km^−2^ yr^−1^	Oxidized nitrogen atmospheric N inputs to regions, 1987
NOY DEP 1992	kg N km^−2^ yr^−1^	Oxidized nitrogen atmospheric N inputs to regions, 1992
NOY DEP 1997	kg N km^−2^ yr^−1^	Oxidized nitrogen atmospheric N inputs to regions, 1997
NOY DEP 2002	kg N km^−2^ yr^−1^	Oxidized nitrogen atmospheric N inputs to regions, 2002
NOY DEP 2007**	kg N km^−2^ yr^−1^	Oxidized nitrogen atmospheric N inputs to regions, 2007
NOY DEP 2012**	kg N km^−2^ yr^−1^	Oxidized nitrogen atmospheric N inputs to regions, 2012
NFIX 1987	kg N km^−2^ yr^−1^	Crop N fixation per area of region, 1987
NFIX 1992	kg N km^−2^ yr^−1^	Crop N fixation per area of region, 1992
NFIX 1997	kg N km^−2^ yr^−1^	Crop N fixation per area of region, 1997
NFIX 2002	kg N km^−2^ yr^−1^	Crop N fixation per area of region, 2002
NFIX 2007	kg N km^−2^ yr^−1^	Crop N fixation per area of region, 2007
NFIX 2012	kg N km^−2^ yr^−1^	Crop N fixation per area of region, 2012
region area	km^2^	region area
TOTAL CROPLAND AREA 1987	km^2^	Total regional cropland area, 1987
TOTAL CROPLAND AREA 1992	km^2^	Total regional cropland area, 1992
TOTAL CROPLAND AREA 1997	km^2^	Total regional cropland area, 1997
TOTAL CROPLAND AREA 2002	km^2^	Total regional cropland area, 2002
TOTAL CROPLAND AREA 2007	km^2^	Total regional cropland area, 2007
TOTAL CROPLAND AREA 2012	km^2^	Total regional cropland area, 2012
Y’, CROP N/TOT AREA 1987	kg N km^−2^ yr^−1^	N content of crops per area of region, 1987
Y’, CROP N/TOT AREA 1992	kg N km^−2^ yr^−1^	N content of crops per area of region, 1992
Y’, CROP N/TOT AREA 1997	kg N km^−2^ yr^−1^	N content of crops per area of region, 1997
Y’, CROP N/TOT AREA 2002	kg N km^−2^ yr^−1^	N content of crops per area of region, 2002
Y’, CROP N/TOT AREA 2007	kg N km^−2^ yr^−1^	N content of crops per area of region, 2007
Y’, CROP N/TOT AREA 2012	kg N km^−^^2^ yr^−1^	N content of crops per area of region, 2012
Y, CROP N/CROPLAND AREA 1987	kg N km^−2^ yr^−1^*	N content of crops per regional cropland area, 1987
Y, CROP N/CROPLAND AREA 1992	kg N km^−2^ yr^−1^*	N content of crops per regional cropland area, 1992
Y, CROP N/CROPLAND AREA 1997	kg N km^−2^ yr^−1^*	N content of crops per regional cropland area, 1997
Y, CROP N/CROPLAND AREA 2002	kg N km^−2^ yr^−1^*	N content of crops per regional cropland area, 2002
Y, CROP N/CROPLAND AREA 2007	kg N km^−2^ yr^−1^*	N content of crops per regional cropland area, 2007
Y, CROP N/CROPLAND AREA 2012	kg N km^−2^ yr^−1^*	N content of crops per regional cropland area, 2012
AG NFERT/CROPLAND AREA, 1987	kg N km^−2^ yr^−1^*	Agricultural fertilizer nitrogen inputs to regions per area of regional cropland, 1987
AG NFERT/CROPLAND AREA, 1992	kg N km^−2^ yr^−1^*	Agricultural fertilizer nitrogen inputs to regions per area of regional cropland, 1992
AG NFERT/CROPLAND AREA, 1997	kg N km^−2^ yr^−1^*	Agricultural fertilizer nitrogen inputs to regions per area of regional cropland, 1997
AG NFERT/CROPLAND AREA, 2002	kg N km^−2^ yr^−1^*	Agricultural fertilizer nitrogen inputs to regions per area of regional cropland, 2002
AG NFERT/CROPLAND AREA, 2007	kg N km^−2^ yr^−1^*	Agricultural fertilizer nitrogen inputs to regions per area of regional cropland, 2007
AG NFERT/CROPLAND AREA, 2012	kg N km^−2^ yr^−1^*	Agricultural fertilizer nitrogen inputs to regions per area of regional cropland, 2012
Pop, 1987	Individuals	Regional population, 1987
Pop, 1992	Individuals	Regional population, 1992
Pop, 1997	Individuals	Regional population, 1997
Pop, 2002	Individuals	Regional population, 2002
Pop, 2007	Individuals	Regional population, 2007
Pop, 2012	Individuals	Regional population, 2012
NFIX/CROPLAND AREA 1987	kg N km^−2^ yr^−1^*	Crop N fixation per regional cropland area, 1987
NFIX/CROPLAND AREA 1992	kg N km^−2^ yr^−1^*	Crop N fixation per regional cropland area, 1992
NFIX/CROPLAND AREA 1997	kg N km^−2^ yr^−1^*	Crop N fixation per regional cropland area, 1997
NFIX/CROPLAND AREA 2002	kg N km^−2^ yr^−1^*	Crop N fixation per regional cropland area, 2002
NFIX/CROPLAND AREA 2007	kg N km^−2^ yr^−1^*	Crop N fixation per regional cropland area, 2007
NFIX/CROPLAND AREA 2012	kg N km^−2^ yr^−1^*	Crop N fixation per regional cropland area, 2012
NEXCR/TOT AREA 1987	kg N km^−2^ yr^−1^	Livestock N excretion per total region area, 1987
NEXCR/TOT AREA 1992	kg N km^−2^ yr^−1^	Livestock N excretion per total region area, 1992
NEXCR/TOT AREA 1997	kg N km^−2^ yr^−1^	Livestock N excretion per total region area, 1997
NEXCR/TOT AREA 2002	kg N km^−2^ yr^−1^	Livestock N excretion per total region area, 2002
NEXCR/TOT AREA 2007	kg N km^−2^ yr^−1^	Livestock N excretion per total region area, 2007
NEXCR/TOT AREA 2012	kg N km^−2^ yr^−1^	Livestock N excretion per total region area, 2012
NEXCR/CROPLAND AREA 1987	kg N km^−2^ yr^−1^*	Livestock N excretion per regional cropland area, 1987
NEXCR/CROPLAND AREA 1992	kg N km^−2^ yr^−1^*	Livestock N excretion per regional cropland area, 1992
NEXCR/CROPLAND AREA 1997	kg N km^−2^ yr^−1^*	Livestock N excretion per regional cropland area, 1997
NEXCR/CROPLAND AREA 2002	kg N km^−2^ yr^−1^*	Livestock N excretion per regional cropland area, 2002
NEXCR/CROPLAND AREA 2007	kg N km^−2^ yr^−1^*	Livestock N excretion per regional cropland area, 2007
NEXCR/CROPLAND AREA 2012	kg N km^−2^ yr^−1^*	Livestock N excretion per regional cropland area, 2012
I, 1987	kg N km^−2^ yr^−1^*	Total ag N inputs per cropland area, 1987
I, 1992	kg N km^−2^ yr^−1^*	Total ag N inputs per cropland area, 1992
I, 1997	kg N km^−2^ yr^−1^*	Total ag N inputs per cropland area, 1997
I, 2002	kg N km^−2^ yr^−1^*	Total ag N inputs per cropland area, 2002
I, 2007	kg N km^−2^ yr^−1^*	Total ag N inputs per cropland area, 2007
I, 2012	kg N km^−2^ yr^−1^*	Total ag N inputs per cropland area, 2012
I’, 1987	kg N km^−2^ yr^−1^	Total ag N inputs per regional area, 1987
I’, 1992	kg N km^−2^ yr^−1^	Total ag N inputs per regional area, 1992
I’, 1997	kg N km^−2^ yr^−1^	Total ag N inputs per regional area, 1997
I’, 2002	kg N km^−2^ yr^−1^	Total ag N inputs per regional area, 2002
I’, 2007	kg N km^−2^ yr^−1^	Total ag N inputs per regional area, 2007
I’, 2012	kg N km^−2^ yr^−1^	Total ag N inputs per regional area, 2012
NUE, 1987	%	Nitrogen Use Efficiency, Y/I, 1987, as %
NUE, 1992	%	Nitrogen Use Efficiency, Y/I, 1992, as %
NUE, 1997	%	Nitrogen Use Efficiency, Y/I, 1997, as %
NUE, 2002	%	Nitrogen Use Efficiency, Y/I, 2002, as %
NUE, 2007	%	Nitrogen Use Efficiency, Y/I, 2007, as %
NUE, 2012	%	Nitrogen Use Efficiency, Y/I, 2012, as %
Nfert%, 1987	%	Ag N fertilizer as % of total regional N input, 1987
Nfert%, 1992	%	Ag N fertilizer as % of total regional N input, 1992
Nfert%, 1997	%	Ag N fertilizer as % of total regional N input, 1997
Nfert%, 2002	%	Ag N fertilizer as % of total regional N input, 2002
Nfert%, 2007	%	Ag N fertilizer as % of total regional N input, 2007
Nfert%, 2012	%	Ag N fertilizer as % of total regional N input, 2012
Nfix%, 1987	%	Crop N fixation as % of total regional N input, 1987
Nfix%, 1992	%	Crop N fixation as % of total regional N input, 1992
Nfix%, 1997	%	Crop N fixation as % of total regional N input, 1997
Nfix%, 2002	%	Crop N fixation as % of total regional N input, 2002
Nfix%, 2007	%	Crop N fixation as % of total regional N input, 2007
Nfix%, 2012	%	Crop N fixation as % of total regional N input, 2012
Nexcr%, 1987	%	Livestock N excr. % of total regional N input, 1987
Nexcr%, 1992	%	Livestock N excr. % of total regional N input, 1992
Nexcr%, 1997	%	Livestock N excr. % of total regional N input, 1997
Nexcr%, 2002	%	Livestock N excr. % of total regional N input, 2002
Nexcr%, 2007	%	Livestock N excr. % of total regional N input, 2007
Nexcr%, 2012	%	Livestock N excr. % of total regional N input, 2012
P 1987***	kg N km^−1^ yr^−1^	Production (N) of livestock products in regions, 1987
LP 1992***	kg N km^−2^ yr^−1^	Production (N) of livestock products in regions, 1992
LP 1997***	kg N km^−2^ yr^−1^	Production (N) of livestock products in regions, 1997
LP 2002***	kg N km^−2^ yr^−1^	Production (N) of livestock products in regions, 2002
LP 2007***	kg N km^−2^ yr^−1^	Production (N) of livestock products in regions, 2007
LP 2012***	kg N km^−2^ yr^−1^	Production (N) of livestock products in regions, 2012

* Indicates cropland area basis instead of total area basis

** N deposition estimate is from the most recent available year of CMAQ model estimates (2006); [1]

*** Beef & veal, pork, poultry, lamb, eggs, milk

## 2 Experimental design, materials, and methods

Briefly, the data contained in the spreadsheets represent components of Net Anthropogenic Nitrogen Input (NANI) and ancillary data obtained using the NANI Calculator Toolbox [1,3] and post processed from these data using simple spreadsheet calculations to calculate variables used in nitrogen use efficiency estimates at county, subregional and regional scales across the continental US. They are based on agricultural production and ancillary data from the US Census of agriculture, population data from the US census, modelled atmospheric nitrogen deposition estimates originating from the USEPA CMAQ model, and a few other sources. Full details on methodology and original sources can be found in the references below.
